# Study on the Influence of mRNA, the Genetic Language, on Protein Folding Rates

**DOI:** 10.3389/fgene.2021.635250

**Published:** 2021-04-06

**Authors:** Ruifang Li, Hong Li, Xue Feng, Ruifeng Zhao, Yongxia Cheng

**Affiliations:** ^1^College of Physics and Electronic Information, Inner Mongolia Normal University, Hohhot, China; ^2^School of Physical Science and Technology, Inner Mongolia University, Hohhot, China

**Keywords:** protein folding rate, genetic language, single base information redundancy, adjacent base related information redundancy, mRNA sequence

## Abstract

Many works have reported that protein folding rates are influenced by the characteristics of amino acid sequences and protein structures. However, few reports on the problem of whether the corresponding mRNA sequences are related to the protein folding rates can be found. An mRNA sequence is regarded as a kind of genetic language, and its vocabulary and phraseology must provide influential information regarding the protein folding rate. In the present work, linear regressions on the parameters of the vocabulary and phraseology of mRNA sequences and the corresponding protein folding rates were analyzed. The results indicated that *D*_2_ (the adjacent base-related information redundancy) values and the GC content values of the corresponding mRNA sequences exhibit significant negative relations with the protein folding rates, but *D*_*1*_ (the single base information redundancy) values exhibit significant positive relations with the protein folding rates. In addition, the results show that the relationships between the parameters of the genetic language and the corresponding protein folding rates are obviously different for different protein groups. Some useful parameters that are related to protein folding rates were found. The results indicate that when predicting protein folding rates, the information from protein structures and their amino acid sequences is insufficient, and some information for regulating the protein folding rates must be derived from the mRNA sequences.

## Introduction

Proteins cannot function properly if they do not fold into their individual structures, and inactive proteins may be produced by misfolding (Price et al., 2018; Wangeline and Hampton, 2018; Jo et al., 2019). Cell deaths or tissue damage may be caused by misfolded proteins (Soto and Pritzkow, 2018; Lee et al., 2020), and misfolded proteins are related to fatal prion diseases (Eraña et al., 2017). It is a great challenge to discover the mechanism of protein folding, and a key step is to find useful factors that are related to protein folding rates. Since 1998, many studies (Plaxco et al., 1998; Mirny and Shakhnovich, 2001; Zhou and Zhou, 2002; Gong et al., 2003; Kuznetsov and Rackovsky, 2004; Punta and Rost, 2005; Choi, 2020; Li et al., 2020,b) have shown that protein folding rates are related to the corresponding protein structures. However, all the above studies required knowledge of the native structures of proteins. There have also been some investigations regarding the prediction of protein folding rates based on amino acid sequences, demonstrating that a protein folding rate depends substantially on the corresponding amino acid sequence (Ivankov and Finkelstein, 2004; Gromiha, 2005; Gromiha et al., 2006; Ouyang and Liang, 2008; Razban, 2019; Szczepaniak et al., 2019).

It is currently believed that many proteins start folding while they synthesize on the ribosome (Komar, 2009; Kemp et al., 2020; Liu, 2020; Walsh et al., 2020) and that mRNA sequences and structures influence the rate of ribosome appearances along mRNA; they then influence the emergence rates of proteins (Razban, 2019). We think that protein folding rates are influenced by the corresponding mRNA sequences in addition to the characteristics of protein structures and amino acid sequences (Li and Li, 2011; Li et al., 2020). mRNA is regarded as a kind of genetic language, and we think that its vocabulary and phraseology must provide some influential information related to protein folding rates. In the present work, we constructed a large dataset and analyzed the relationships between the parameters of genetic language and protein folding rates to determine the influence of mRNA. We determined that protein folding rate is also influenced by the corresponding mRNA sequence in addition to the characteristics of amino acid sequence and protein structure. If we can add the influential factors of mRNA sequences into the protein folding rate prediction, its accuracy would be greatly improved.

## Materials

### Dataset

In recent years, some experimental data on protein folding rates had been reported, Ouyang and Liang (2008) developed a method that could predict the folding rates for proteins based on the amino acid sequences of 80 proteins. Ivankov et al. (2009) studied the coupling between properties of the protein shape and the rate of protein folding based on a dataset of 84 proteins. Guo et al. (2011) predicted folding rates of 99 proteins. But information on the corresponding mRNA sequences not contained within such datasets. In the present work, we collected these data, eliminated redundant data and found information regarding the corresponding mRNA sequence of each protein. Finally, we constructed a new dataset containing 100 proteins, of which 56 are two-state folders (proteins that could fold rapidly without populating any intermediate states) and 44 are multistate folders (proteins that fold to their native states via a populated intermediate state), and according to their structural classifications, they were divided into three groups (21 are all-α proteins, 39 are all-β proteins, and 40 are α-β proteins). It should be noted that the values of protein folding rates vary greatly from a few microseconds to several hours. So, in order to compare them in a table or a figure, the natural logarithm of protein folding rate [ln*(k*_*f*_*)*] was usually used to represent protein folding rate in previous studies. In the present study, we also defined the value of protein folding rate with its natural logarithm.

### Amino Acid Sequences and Their Corresponding mRNA Sequences

The corresponding mRNA sequences of the proteins were taken from the European Molecular Biology Laboratory (EMBL) through cross-referencing with the Protein Data Bank (PDB). Some of the proteins were protein segments, so we intercepted these protein sequences and their corresponding mRNA sequences. Information about the 100 proteins and segments is given in Supplementary Appendix Table 1.

## Methods

### mRNA Properties

From the related studies, we learned that the properties extracted from 3D structures and the primary sequences of proteins are very useful for predicting their folding rates. However, we think the above properties are not enough for such predictions; here, let us focus on the properties derived from mRNA sequences. The basic information of an mRNA sequence is its base composition and the base relations, which represent the vocabulary and phraseology of the genetic language, respectively. Luo observed that the base relations are mainly embodied in the adjacent relations and proposed some parameters (Luo et al., 1998), such as the single base information redundancy (*D*_1_), the adjacent base related information redundancy (*D*_2_), and two other parameters derived from, *D*_1_ and *D*_2_. All these parameters were proven to be related to evolution. In the present work, we selected the GC content of mRNA sequences, *D*_1_ and *D*_2_, which represent the information regarding the genetic language of the mRNA sequence to analyze the relations between mRNA sequence and protein folding rate. The parameters are described in detail as follows:

### Single Base Information Redundancy

An RNA sequence is a kind of genetic language; *D*_1_is the single base information redundancy, which was introduced to describe the composition of the vocabulary of the genetic language, and it indicates the differences in the base distributions between the observed sequence and a random sequence. It can be calculated by equation (1).

(1) $$D_{1}=2+\sum_{i}p_{i}\log_{2}p_{i}$$

where *D*_1_is the single base information redundancy and *p*_*i*_ is the probability of base *i* (*i* = A, U, G or C).

### Adjacent Base Related Information Redundancy

mRNA sequences contain much information, most of which is contained in the base correlation, especially in the adjacent base correlation. *D*_2_ is the adjacent base related information redundancy, which was introduced to describe the phraseology of the genetic language. *D*_2_ can be calculated by equation (2).

(2) $$D_{2}=-2\sum_{i}p_{i}\log_{2}p_{i}+\sum_{i,j}p_{ij}\log_{2}p_{ij}$$

(3) $$p_{ij}=p_{i}p_{j|i}$$

where *D*_2_ is the adjacent base related information redundancy, *p*_*i*_ is the probability of base *i* (*i* = A, U, G or C), *p*_*ij*_is the probability of dinucleotide *ij*, and *p*_*j|i*_ is conditional probability of base *j* occurred after base *i*.

### GC Content

In the present work, another derived parameter is the GC content, which can be calculated by equation (4).

(4) $$C_{GC}=\left(N_{G}+N_{C}\right)/N$$

where *C*_*GC*_ is the GC content of an mRNA sequence, *N*_*G*_ and *N*_*C*_ are the amounts of base G and base C, respectively, and *N* is the total base number of the mRNA sequence.

### The Information Parameters of Subsequences

An increasing number of people are realizing the differences between the 3 positions of a codon. For the mRNA sequence of each protein, we picked out all the nucleotides in the first positions of the codons in the sequence and made a new sequence. The new sequence was named subsequence 1, and likewise, we obtained subsequence 2 and subsequence 3. Then, we defined the corresponding parameters of each subsequence according to equations (1), (2), (3) and (4). They are: *$D_{1}^{1}$*, $D_{2}^{1}$,$C_{GC}^{1}$, $D_{1}^{2}$, $D_{2}^{2}$, $C_{GC}^{2}$, $D_{1}^{3}$, $D_{2}^{3}$ and $C_{GC}^{3}$.

The values of the above parameters for each protein were calculated, and the values are shown in Supplementary Appendix Table 2.

### Linear Regression Procedures

First, for all 100 proteins, we performed linear regression analysis on the values of each parameter (*C*_*GC*_, *D*_1_, *D*_2_, $D_{1}^{1}$, $D_{2}^{1}$, $C_{GC}^{1}$, $D_{1}^{2}$, $D_{2}^{2}$, $C_{GC}^{2}$, $D_{1}^{3}$, $D_{2}^{3}$ and $C_{GC}^{3}$) and the experimental protein folding rates. Second, we performed the same linear regression analysis separately for 56 two-state folders and 44 multistate folders. Finally, the same linear regression analysis was performed separately for 21 all-α proteins, 39 all-β proteins, and 40 α-β proteins. Then, we verified the statistical significance of the regression models with their *p*-values.

## Results

### The Correlations of all the 100 Proteins

According to the above discussion, we selected 12 properties extracted from the mRNA sequences. Each of these properties may be correlated with protein folding rates. First, for all 100 proteins, linear regression analyses were performed on the values of each parameter and the protein folding rates. Previous related works demonstrated that two-state folders and multistate folders represent different features in terms of predicting protein folding rates. Second, we divided the proteins into two-state proteins and multistate proteins, and then, the same linear regression analyses were performed for each type of protein. The results are presented in Table 1.

**TABLE 1:** Results of linear regression between the protein folding rates and the parameters of the corresponding mRNA sequences of the 100 proteins.

	**C*_*GC*_*	**D*_*1*_*	**D*_*2*_*	*$C_{GC}^{1}$*	*$D_{1}^{1}$*	*$D_{2}^{1}$*	*$C_{GC}^{2}$*	*$D_{1}^{2}$*	*$D_{2}^{2}$*	*$C_{GC}^{3}$*	*$D_{1}^{3}$*	*$D_{2}^{3}$*
All	−0.19^∗^	0.29^∗∗^	–0.23^∗∗^	–0.28^∗∗^	0.11	–0.04	–0.05	0.33^∗∗∗^	–0.29^∗∗^	–0.12	0.08	–0.08
Two-state	–0.09	0.23	–0.19	–0.09	0.07	–0.06	–0.17	0.40^∗∗^	–0.37^∗∗^	–0.02	0.12	–0.10
Multi-state	−0.34^∗^	0.24	−0.28^∗^	–0.41^∗∗^	0.13	0.09	0.09	0.14	–0.06	−0.32^∗^	0.02	–0.01

*Note: the numbers are the correlation coefficients, Two-tailed significance: the symbol “^∗^” means *P* < 0.05, the symbol “^∗∗^” means *P* < 0.01, and the symbol “^∗∗∗^” means *P* < 0.001.*

**FIGURE 1:**
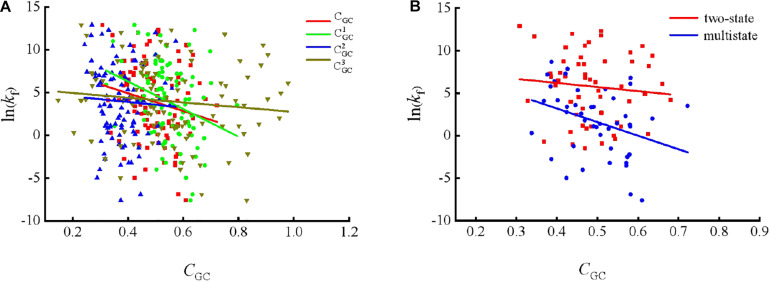
Changes of protein folding rates with the values of GC content in mRNA. **(A)** Changes of protein folding rates with the values of GC content (*C*_*GC*_,$C_{GC}^{1}$, $C_{GC}^{2}$ and $C_{GC}^{3}$) in mRNA of all the 100 proteins. **(B)** Changes of protein folding rates with the values of GC content (*C*_*GC*_) in mRNA of the two-state folders and multistate folders.

**FIGURE 2:**
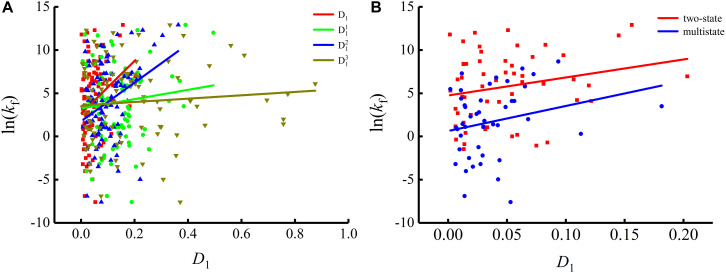
Changes of protein folding rates with the values of the single base information redundancy in mRNA. **(A)** Changes of protein folding rates with the values of the single base information redundancy (*D*_*1*_, $D_{1}^{1}$, $D_{1}^{2}$ and $D_{1}^{3}$) in mRNA of all the 100 proteins. **(B)** Changes of protein folding rates with the values of the single base information redundancy (*D*_*1*_) in mRNA of the two-state folders and multistate folders.

**FIGURE 3:**
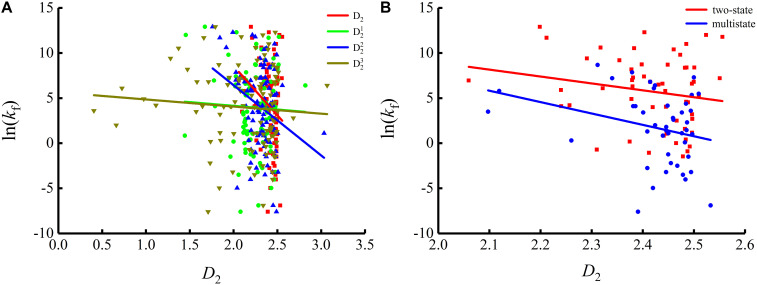
Changes of protein folding rates with the values of the adjacent base related information redundancy in mRNA. **(A)** Changes of protein folding rates with the values of the adjacent base related information redundancy (*D*_*2*_, $D_{2}^{1}$, $D_{2}^{2}$ and $D_{2}^{3}$) in mRNA of all the 100 proteins. **(B)** Changes of protein folding rates with the values of the adjacent base related information redundancy (*D*_*2*_) in mRNA of the two-state folders and multistate folders.

To show the correlations between the parameters of the corresponding mRNA sequences and the protein folding rates clearly, we drew figures of the protein folding rates along with their corresponding parameters (see Figures 1–3).

As we can see from Table 1 and Figure 1, the parameter *C*_*GC*_ is negatively correlated with the protein folding rates, and further analysis showed that the effect of GC content on the protein folding rates is mainly derived from the first and third positions of the codons. The parameter *D*_1_ is positively related to the protein folding rates, and we found that the parameters $D_{1}^{2}$ is strongly and positively related to the protein folding rates, this phenomenon is shown in Table 1 and Figure 2. Parameter *D*_2_ is negatively correlated with the protein folding rates. In addition, parameter $D_{2}^{2}$ exhibited significant negative relations with the protein folding rates. In addition, parameter $D_{2}^{2}$ exhibited significant positive relations with the protein folding rates. Multistate folders yielded the highest correlation coefficients, reaching 0.44, as shown in Table 1 and Figure 3. At the same time, we noticed that the correlation was more significant for multistate folders than for two-state folders, and this can be seen in Table 1, Figures 1–3. Our results indicated that increasing the GC content and the *D*_*2*_ values may hinder the protein folding process, and increasing the *D*_*1*_ values may enhance the protein folding process; however, the influence of parameter *D*_1_ on the two-state folders is the opposite of its influence on the multistate folders. The results proved that the protein folding rates are also influenced by the vocabulary and phraseology of mRNA sequences.

### The Correlations of Proteins in Different Structural Classes

In previously published related work, it was found that the valid parameters for predicting protein folding rates are distinct for different structural classes. Therefore, it is necessary to classify the proteins into different structural classes. In the present work, we divided the 100 proteins into groups of all-α proteins, all-β proteins, and α-β proteins. In each group, we performed the same regression analyses as in the above section, and the results are presented in Tables 2–4.

**TABLE 2:** Results of linear regression between the protein folding rates and the parameters of the corresponding mRNA sequences of the 21 all-*α*proteins.

	**C*_*GC*_*	**D*_*1*_*	**D*_*2*_*	*$C_{GC}^{1}$*	*$D_{1}^{1}$*	*$D_{2}^{1}$*	*$C_{GC}^{2}$*	*$D_{1}^{2}$*	*$D_{2}^{2}$*	*$C_{GC}^{3}$*	*$D_{1}^{3}$*	*$D_{2}^{3}$*
All	–0.28	–0.05	0.20	–0.32	–0.08	–0.09	–0.15	0.34^∗^	−0.33^∗^	–0.21	–0.04	–0.07
Two-state	–0.05	0.02	0.08	0.19	0.22	–0.24	–0.22	0.39	–0.37	–0.07	0.21	–0.22
Multi-state	−0.75^∗^	0.02	–0.02	–0.69	–0.21	–0.01	–0.20	0.44	–0.48	−0.80^∗^	–0.57	–0.24

*Note: the numbers are the correlation coefficients, Two-tailed significance: the symbol “^∗^” means *P* < 0.05, the symbol “^∗∗^” means *P* < 0.01, and the symbol “^∗∗∗^” means *P* < 0.001.*

**TABLE 3:** Results of linear regression between the protein folding rates and the parameters of the corresponding mRNA sequences of the 39 all-*β* proteins.

	**C*_*GC*_*	**D*_*1*_*	**D*_*2*_*	*$C_{GC}^{1}$*	*$D_{1}^{1}$*	*$D_{2}^{1}$*	*$C_{GC}^{2}$*	*$D_{1}^{2}$*	*$D_{2}^{2}$*	*$C_{GC}^{3}$*	*$D_{1}^{3}$*	*$D_{2}^{3}$*
All	–0.10	0.15	–0.09	–0.19	0.29	−0.29^∗^	0.17	0.14	–0.13	–0.13	–0.03	–0.04
Two-state	0.01	0.18	–0.13	–0.04	0.24	–0.27	–0.07	0.43^∗^	−0.37^∗^	0.05	0.10	–0.05
Multi-state	–0.21	0.45	0.46	–0.48	0.37	–0.41	0.35	–0.31	0.12	–0.25	–0.51	0.05

*Note: the numbers are the correlation coefficients, Two-tailed significance: the symbol “^∗^” means *P* < 0.05, the symbol “^∗∗^” means *P* < 0.01, and the symbol “^∗∗∗^” means *P* < 0.001.*

**TABLE 4:** Results of linear regression between the protein folding rates and the parameters of the corresponding mRNA sequences of the 40*α*-*β* proteins.

	**C*_*GC*_*	**D*_*1*_*	**D*_*2*_*	*$C_{GC}^{1}$*	*$D_{1}^{1}$*	*$D_{2}^{1}$*	*$C_{GC}^{2}$*	*$D_{1}^{2}$*	*$D_{2}^{2}$*	*$C_{GC}^{3}$*	*$D_{1}^{3}$*	*$D_{2}^{3}$*
All	–0.16	0.23	–0.22	−0.35^∗^	–0.12	0.28	−0.30^∗^	0.39^∗∗^	–0.21	0.02	0.26	–0.23
Two-state	–0.01	0.08	–0.04	0.06	–0.32	0.38	–0.48	0.31	–0.34	0.13	0.26	–0.25
Multi-state	–0.33	0.24	–0.31	–0.60^∗∗∗^	–0.05	0.30	–0.09	0.34	–0.01	–0.17	0.22	–0.16

*Note: the numbers are the correlation coefficients, Two-tailed significance: the symbol“^∗^” means *P* < 0.05, the symbol “^∗∗^” means *P* < 0.01, and the symbol “^∗∗∗^” means *P* < 0.001.*

As we hypothesized, the results are different for different protein groups. For example, GC content has different influences on proteins in different structural classes. In detail, the influence of parameter *C*_*GC*_ is mainly derived from the third positions of the codons for all-α proteins, but it is mainly derived from the first positions of the codons for α-β proteins, and parameter *C*_*GC*_ has little influence on the protein folding rates for all-β proteins. In addition, we noticed that for all-α multistate folders, parameter $C_{GC}^{3}$ exhibited significant correlations with the protein folding rates, yielding the highest correlation coefficient (reaching 0.80). This indicates that this kind of effect mostly comes from synonymous codon usage and not from the information of amino acids.

Of course, some results were the same for different protein groups. For example, the parameter $D_{1}^{2}$ exhibited an excellent positive relations with the folding rates of each structural class. Furthermore, parameter $D_{2}^{2}$ exhibited significant negative relations with the folding rates of all-*α* proteins and *β* proteins. In addition, it is obvious that the correlations are more significant for multistate folders in each structural class than for two-state folders.

The mRNA sequence and its subsequences are regarded as genetic language. The above results indicate that both the vocabulary and phraseology of mRNA may influence the corresponding protein folding rate, and parameters such as $C_{GC}^{3}$, $D_{1}^{1}$ and $D_{2}^{2}$ may be influential parameters for protein folding rate prediction.

## Discussion

In theory, mRNA structures may be influenced by the vocabulary and phraseology of their mRNA sequences. In detail, the complexity and variability of mRNA secondary or higher structures are determined partly by the base relations in the mRNA sequence. We think that mRNA structures must influence the rate of ribosome appearances along mRNA; and then influence the emergence rates of proteins, and we also think that the base relations are mainly embodied in adjacent relations. Therefore, the two parameters (single base information redundancy and adjacent base related information redundancy) provide information regarding the variability and complexity of mRNA structures, and the results show that the above two parameters may be effective factors for predicting protein folding rates.

It is interesting that for the multistate folders, the influence of GC content is outstanding. In detail, for all-α proteins, the influence of parameter *C*_*GC*_ is mainly derived from the third positions of the codons, but for α-β proteins, it is mainly derived from the first positions of the codons. The composition of the second codon position is incredibly stable, with very little deviation in composition across the species, but the composition of the third codon position has a large deviation because of the bias of the synonymous codon usage (Gibson et al., 2005). We think that the large deviation of base composition results in a large range of regulating, Therefore, the effect of GC content on the protein folding rates is mainly derived from the first and third positions of the codons.

The influence of the third positions of codons is inspiring because the third positions take information regarding synonymous codon bias but not amino acid bias. This means that this part of the information is only obtained from the mRNA sequence, not from amino acids. This additionally proves that the folding rates are also influenced by the non-random usage of synonymous codons.

## Conclusion

To conclude, in this work, some parameters of the vocabulary and phraseology of mRNA sequences were selected, and then, the relationships of these parameters with protein folding rates were analyzed. The results showed that the vocabulary and phraseology of mRNA sequences are significantly correlated with protein folding rates to different degrees. This suggests that the evaluated mRNA sequence plays an important role in regulating protein folding.

Although our parameters are simple parameters for representing mRNA information, their influences are significant. If we can find better parameters to represent the mRNA information, we believe that more detailed and clearer relations between mRNA sequences and protein folding rates will be discovered.

## Data Availability Statement

The original contributions presented in the study are included in the article/Supplementary Material, further inquiries can be directed to the corresponding author/s.

## Author Contributions

RFL, HL, XF, RFZ, and YXC performed the work. RFL has done the work of the topic selection and the data analysis, and wrote the manuscript. HL has took part in the work of the theoretical analysis. XF, RFZ, and YXC performed the work calculation. All authors have read and approved this version of the article, and due care has been taken to ensure the integrity of the work.

## Conflict of Interest

The authors declare that the research was conducted in the absence of any commercial or financial relationships that could be construed as a potential conflict of interest.
