# Supplementary material for: Study on the Influence of mRNA, the Genetic Language, on Protein Folding Rates
Source: Front Genet. 2021 Apr 6;12:635250. doi: 10.3389/fgene.2021.635250 (PMC8056030; doi:10.3389/fgene.2021.635250)
Supplement: Supplementary file 1 [file Table_1.pdf]

## APPENDIX TABLE 1| The set of 100 proteins

| Two-state | | | | Multistate | | | |
|---|---|---|---|---|---|---|---|
| PDB ID | Length | ln($k_f$) | Structural class | PDB ID | length | ln($k_f$) | Structural class |
| 1ARR | 53 | 9.20 | *α* | 1A6N | 151 | 1.10 | *α* |
| 1BA5 | 53 | 5.90 | *α* | 1AYI | 85 | 7.20 | *α* |
| 1BDD | 60 | 11.69 | *α* | 1CEI | 85 | 5.8 | *α* |
| 1ENH | 54 | 10.53 | *α* | 1UZC | 69 | 8.68 | *α* |
| 1EFX | 59 | 8.19 | *α* | 2A5E | 127 | 3.50 | *α* |
| 1IDY | 54 | 8.73 | *α* | 2ABD | 86 | 7.86 | *α* |
| 1IMQ | 85 | 7.28 | *α* | 2CRO | 65 | 5.35 | *α* |
| 1LMB | 80 | 10.4 | *α* | 1ADW | 123 | -7.60 | *β* |
| 1NTI | 86 | 7.00 | *α* | 1B9C | 224 | -2.76 | *β* |
| 1PRB | 47 | 12.9 | *α* | 1BEB | 156 | -2.20 | *β* |
| 1U5P | 110 | 11.0 | *α* | 1CBI | 136 | -3.20 | *β* |
| 1VII | 36 | 11.8 | *α* | 1EAL | 127 | 1.30 | *β* |
| 256B | 106 | 12.3 | *α* | 1FNF-10 | 94 | 5.48 | *β* |
| 2PDD | 41 | 9.80 | *α* | 1HCD | 118 | -4.97 | *β* |
| 1C8C | 64 | 6.95 | *β* | 1HNG | 95 | 1.80 | *β* |
| 1C9O | 66 | 7.20 | *β* | 1I1B | 151 | -4.01 | *β* |
| 1CSP | 67 | 6.50 | *β* | 1IFC | 131 | 3.40 | *β* |
| 1E65 | 128 | 4.91 | *β* | 1OPA | 133 | 1.40 | *β* |
| 1E0L | 37 | 10.6 | *β* | 1TIT | 89 | 3.60 | *β* |
| 1FMK | 57 | 4.05 | *β* | 1AON | 155 | -1.50 | *α-β* |
| 1FNF-9 | 90 | -0.90 | *β* | 1BNI | 108 | 2.6 | *α-β* |
| 1G6P | 66 | 6.30 | *β* | 1BRS | 89 | 3.40 | *α-β* |
| 1JMQ | 40 | 8.40 | *β* | 1BTA | 89 | 1.11 | *α-β* |

| | | | | | | | |
|---|---|---|---|---|---|---|---|
| 1JO8 | 58 | 2.50 | *β* | 1DK7 | 146 | 0.83 | *α-β* |
| 1K0S | 143 | 7.40 | *β* | 1FKB | 96 | 1.45 | *α-β* |
| 1K8M | 87 | -0.71 | *β* | 1GXT | 88 | 4.39 | *α-β* |
| 1K9Q | 40 | 8.37 | *β* | 1HEL | 129 | 6.10 | *α-β* |
| 1LOP | 164 | 6.6 | *β* | 1HMK | 121 | 2.79 | *α-β* |
| 1M9S | 76 | 4.00 | *β* | 1JOO | 149 | 0.30 | *α-β* |
| 1MJC | 69 | 5.30 | *β* | 1L63 | 162 | 4.1 | *α-β* |
| 1NYF | 58 | 4.54 | *β* | 1N88 | 96 | 2.00 | *α-β* |
| 1PGB-B | 16 | 12.0 | *β* | 1PGB | 56 | 6.4 | *α-β* |
| 1PIN | 34 | 9.40 | *β* | 1PHP-N | 175 | 2.30 | *α-β* |
| 1PKS | 76 | -1.06 | *β* | 1PHP-C | 219 | -3.50 | *α-β* |
| 1PSE | 69 | 1.17 | *β* | 1QOP-A | 269 | -2.50 | *α-β* |
| 1PSF | 69 | 3.2 | *β* | 1QOP-B | 390 | -6.90 | *α-β* |
| 1QTU | 108 | -0.36 | *β* | 1RA9 | 159 | -3.20 | *α-β* |
| 1SHG | 57 | 1.10 | *β* | 1SCE | 97 | 4.17 | *α-β* |
| 1TEN | 89 | 1.06 | *β* | 1UBQ | 76 | 7.30 | *α-β* |
| 1WIT | 93 | 0.41 | *β* | 2BLM | 260 | -1.24 | *α-β* |
| 2AIT | 74 | 4.21 | *β* | 2LZM | 164 | 4.10 | *α-β* |
| 1APS | 98 | -1.47 | *α-β* | 2RN2 | 155 | 0.10 | *α-β* |
| 1DIV | 56 | 6.6 | *α-β* | 2VIK" | 126 | 6.80 | *α-β* |
| 1DIV-C | 92 | 0.0 | *α-β* | 3CHY | 128 | 1.00 | *α-β* |
| 1FKF | 107 | 1.60 | *α-β* | | | | |
| 1HDN | 85 | 2.69 | *α-β* | | | | |
| 1O6X | 71 | 6.80 | *α-β* | | | | |
| 1RFA | 78 | 8.40 | *α-β* | | | | |
| 1RIS | 97 | 6.10 | *α-β* | | | | |
| 1SPR | 103 | 8.70 | *α-β* | | | | |
| 1URN | 96 | 4.60 | *α-β* | | | | |

| | | | |
|---|---|---|---|
| 2ACY | 98 | 0.84 | *α-β* |
| 2CI2 | 64 | 5.80 | *α-β* |
| 2HQI | 72 | 0.18 | *α-β* |
| 2PTL | 60 | 4.10 | *α-β* |
| 2VIK | 126 | 6.80 | *α-β* |

Note: $\ln(k_f)$ is the folding rate of the protein.
