# Supplementary material for: Study on the Influence of mRNA, the Genetic Language, on Protein Folding Rates
Source: Front Genet. 2021 Apr 6;12:635250. doi: 10.3389/fgene.2021.635250 (PMC8056030; doi:10.3389/fgene.2021.635250)
Supplement: Supplementary file 2 [file Table_2.pdf]

APPENDIX TABLE2 | the values of parameters for the 100 proteins

PDB ID	C_{GC}	D_1	D_2	C_{GC}^1	D_1^1	D_2^1	C_{GC}^2	D_1^2	D_2^2	C_{GC}^3	D_1^3	D_2^3
1ARR	0.4591	0.0718	2.3544	0.5094	0.0759	2.3617	0.3585	0.0961	2.3661	0.5094	0.1204	2.2691
1BA5	0.3962	0.1196	2.2399	0.3208	0.1706	2.2040	0.3774	0.1489	2.2000	0.4906	0.0971	2.3107
1BDD	0.3444	0.1450	2.2118	0.5167	0.0620	2.3996	0.2667	0.3045	1.8437	0.2500	0.3830	1.6610
1ENH	0.6111	0.0588	2.3830	0.5370	0.0710	2.3735	0.4074	0.0740	2.3742	0.8889	0.5601	1.3721
1FEX	0.6271	0.0543	2.3779	0.5424	0.0227	2.5014	0.4576	0.0304	2.4682	0.8814	0.4801	1.4752
1IDY	0.5185	0.0487	2.3981	0.5000	0.1224	2.2475	0.4259	0.0698	2.3504	0.6296	0.0580	2.4527
1IMQ	0.3837	0.0560	2.3811	0.5116	0.0906	2.3073	0.3721	0.0853	2.3135	0.2674	0.1752	2.1088
1LMB	0.4215	0.0267	2.4605	0.5402	0.0863	2.3207	0.3448	0.0839	2.3473	0.3793	0.0593	2.3927
1NTI	0.4205	0.0631	2.3793	0.5114	0.1659	2.1429	0.2955	0.2197	2.0264	0.4545	0.0133	2.5120
1prb	0.3077	0.1561	2.1987	0.4231	0.3905	1.6735	0.2692	0.3634	1.7607	0.2308	0.3322	1.7924
1U5P	0.4788	0.0127	2.4852	0.3455	0.1132	2.2595	0.5727	0.0231	2.4676	0.5182	0.0488	2.4030
1VII	0.5093	0.0015	2.5558	0.4722	0.0139	2.5165	0.3056	0.1359	2.2467	0.7500	0.2158	2.0886
256B	0.5094	0.0509	2.4032	0.6415	0.2106	2.0332	0.3302	0.2307	1.9922	0.5566	0.0193	2.4754
2PDD	0.5814	0.0281	2.4734	0.6977	0.2584	1.9419	0.4186	0.0154	2.4928	0.6279	0.0933	2.3534
1A6N	0.5806	0.0299	2.4500	0.6225	0.1239	2.2146	0.3179	0.1214	2.2950	0.8013	0.2989	2.2297
1AYI	0.3765	0.0725	2.3403	0.5765	0.1758	2.1199	0.2824	0.1835	2.1126	0.2706	0.2031	2.0660
1CEI	0.3843	0.0652	2.1190	0.5765	0.1758	2.4543	0.3059	0.1630	2.1560	0.2706	0.1859	2.1009
1UZC	0.3865	0.0936	2.3116	0.3768	0.1242	2.2522	0.4203	0.1004	2.3142	0.3623	0.2064	2.0563
2A5E	0.7222	0.1815	2.0974	0.7949	0.3840	2.1538	0.5513	0.0090	2.4890	0.8205	0.4335	1.5744
2ABD	0.4264	0.0631	2.3793	0.5233	0.1659	2.1429	0.3023	0.2197	2.0264	0.4535	0.0133	2.5120
2CRO	0.4513	0.0109	2.4958	0.5231	0.0458	2.1533	0.4000	0.0448	2.4394	0.4308	0.0411	2.4222
1C8C	0.3854	0.2033	2.0595	0.4688	0.3410	1.7772	0.3125	0.1619	2.1945	0.3750	0.2119	2.0602
1C90	0.4697	0.0300	2.5506	0.5909	0.1580	1.9460	0.3182	0.1168	2.3023	0.5000	0.1182	2.2566
1CSP	0.403	0.0407	2.4307	0.5970	0.2253	2.0234	0.3384	0.0928	2.3226	0.2836	0.2593	1.9338

1E65	0.6172	0.0543	2.3780	0.5313	0.0765	2.3357	0.3672	0.0669	2.3539	0.9531	0.7729	0.9756
1E0L	0.4685	0.0827	2.3174	0.6216	0.1183	2.2553	0.2432	0.2735	1.9165	0.5405	0.1530	2.2509
1FMK	0.5789	0.0331	2.4453	0.4912	0.0276	2.4766	0.4561	0.0434	2.4096	0.7895	0.2847	1.8876
1FNF-9	0.5111	0.0138	2.4876	0.5333	0.0420	2.4260	0.5111	0.0683	2.4829	0.4889	0.1340	2.2148
1G6P	0.4949	0.0630	2.3799	0.5758	0.2209	2.0188	0.3030	0.1391	2.2268	0.6060	0.0967	2.2981
1JMQ	0.4667	0.0131	2.5009	0.5000	0.0181	2.5031	0.4500	0.0739	2.4084	0.4500	0.0440	2.4907
1JO8	0.4483	0.0193	2.4662	0.5517	0.1773	2.1306	0.3103	0.1547	2.2214	0.4828	0.0313	2.4617
1K0S	0.4079	0.0489	2.4025	0.5245	0.2115	2.0263	0.2517	0.1903	2.0770	0.4476	0.0144	2.4787
1K8M	0.3682	0.0843	2.3100	0.5349	0.1788	2.1354	0.3023	0.1436	2.1991	0.2674	0.1504	2.1807
1K9Q	0.4667	0.0131	2.4825	0.5000	0.0181	2.5031	0.4500	0.0739	2.4084	0.4500	0.0440	2.4907
1LOP	0.4817	0.0080	2.4962	0.5427	0.1497	2.1704	0.3476	0.0732	2.3644	0.5549	0.0842	2.3202
1M9S	0.4342	0.0432	2.4195	0.4868	0.0513	2.4062	0.4342	0.0507	2.4077	0.3816	0.1228	2.2379
1MJC	0.5024	0.0019	2.5098	0.5072	0.1611	2.1393	0.4203	0.0259	2.4684	0.5797	0.1317	2.2070
1NYF	0.454	0.0139	2.4887	0.5345	0.0738	2.3704	0.4138	0.0369	2.4470	0.4138	0.0251	2.4746
1PGB-B	0.4375	0.0228	2.5238	0.4375	0.4948	1.4547	0.5000	0.1887	2.2143	0.3750	0.1198	2.3342
1PIN	0.6354	0.0980	2.2907	0.5000	0.0430	2.4563	0.5000	0.1021	2.3022	0.9063	0.6105	1.2809
1PKS	0.4386	0.0750	2.4122	0.5263	0.1804	2.1364	0.3684	0.1242	2.2499	0.4211	0.0297	2.4550
1PSE	0.4638	0.0063	2.4963	0.5217	0.1794	2.1132	0.3768	0.0726	2.3435	0.4928	0.2955	1.8455
1PSF	0.4638	0.0063	2.4963	0.5217	0.1794	2.1132	0.3768	0.0726	2.3435	0.4928	0.2955	1.8455
1QTU	0.5432	0.0067	2.4918	0.6759	0.0915	2.4222	0.3426	0.0775	2.3502	0.6111	0.0431	2.3692
1SHG	0.4854	0.0299	2.4487	0.3509	0.1420	2.2671	0.5088	0.0079	2.5031	0.5965	0.1165	2.2714
1TEN	0.5581	0.0332	2.4466	0.5169	0.1299	2.2172	0.4157	0.0842	2.3273	0.7416	0.2259	2.0021
1WIT	0.4337	0.0148	2.4841	0.4946	0.1400	2.1939	0.4194	0.0653	2.3719	0.3871	0.0490	2.4009
2AIT	0.6802	0.1095	2.2567	0.6216	0.0867	2.3365	0.5135	0.0273	2.4487	0.9054	0.6951	1.1150
1ADW	0.6098	0.0530	2.3905	0.6260	0.1892	2.0785	0.3740	0.0754	2.4898	0.8293	0.3708	1.7102
1B9C	0.3862	0.0436	2.4090	0.5242	0.0775	2.3285	0.3172	0.1113	2.2604	0.3172	0.1301	2.1813
1BEB	0.5791	0.0293	2.4552	0.5897	0.0866	2.3018	0.3141	0.1335	2.1963	0.8333	0.3551	1.7321
1CBI	0.5711	0.0276	2.4456	0.5368	0.1122	2.2736	0.4118	0.0325	2.5085	0.7647	0.2299	1.9903

1EAL	0.5801	0.0422	2.4090	0.5118	0.1510	2.1651	0.3543	0.0859	2.3194	0.874	0.4636	2.4960
1FNF-10	0.5197	0.0019	2.5095	0.5161	0.1540	2.1534	0.5591	0.0184	2.4900	0.4839	0.0762	2.3350
1HCD	0.4237	0.0424	2.4199	0.6102	0.0404	2.4306	0.2881	0.2210	2.0407	0.3729	0.3074	1.8379
1HNG	0.4688	0.0308	2.4459	0.4583	0.1727	2.1104	0.3854	0.0428	2.4453	0.5625	0.0317	2.4377
1I1B	0.4636	0.0151	2.4837	0.5033	0.0200	2.4616	0.2980	0.1387	2.1928	0.5894	0.0344	2.4467
1IFC	0.4249	0.0488	2.4008	0.4962	0.1747	2.1101	0.2977	0.1346	2.2181	0.4809	0.0048	2.4977
1OPA	0.5063	0.0383	2.4462	0.5263	0.1228	2.1283	0.3233	0.1375	2.2008	0.6692	0.0912	2.3807
1TIT	0.4457	0.0140	2.4939	0.6067	0.0975	2.2945	0.3146	0.1119	2.2788	0.4157	0.0448	0.4057
1APS	0.4694	0.0132	2.4785	0.4388	0.1187	2.2280	0.4388	0.0214	2.4804	0.5306	0.0172	2.4791
1DIV	0.4167	0.1067	2.4385	0.5179	0.1925	2.1361	0.2679	0.2079	2.0538	0.4643	0.1522	2.4745
1DIV-C	0.5399	0.0544	2.4652	0.6304	0.1677	2.1243	0.3696	0.1098	2.2804	0.6196	0.1457	2.3425
1FKF	0.5483	0.0132	2.4825	0.6168	0.0882	2.3167	0.4206	0.0202	2.4609	0.6075	0.0368	2.4214
1HDN	0.5098	0.0082	2.4970	0.6000	0.1641	2.1362	0.4000	0.0810	2.3328	0.5294	0.0111	2.4872
1O6X	0.4695	0.0116	2.4998	0.6338	0.0776	2.3472	0.2817	0.1775	2.1262	0.4930	0.0247	2.4620
1RFA	0.453	0.0153	2.4856	0.5513	0.0507	2.4171	0.3462	0.0782	2.3419	0.4615	0.0052	2.5021
1RIS	0.6598	0.0811	2.3217	0.7010	0.1522	2.1800	0.2990	0.1304	2.2072	0.9794	0.8758	0.7258
1SPR	0.5858	0.0269	2.4488	0.5243	0.0058	2.5132	0.4272	0.0304	2.4416	0.8058	0.3262	1.8334
1URN	0.5069	0.0119	2.4850	0.4583	0.0779	2.3431	0.3229	0.1134	2.2703	0.7396	0.2343	2.0160
2ACY	0.4728	0.0316	2.4410	0.5714	0.0745	2.3568	0.3265	0.1057	2.2756	0.5204	0.0372	2.4366
2CI2	0.559	0.0291	2.4441	0.6308	0.1794	2.0972	0.2923	0.1302	2.2081	0.7538	0.2152	2.0769
2HQI	0.625	0.0588	2.3745	0.5833	0.2316	2.0001	0.4722	0.0628	2.3855	0.8194	0.3581	1.7365
2PTL	0.3279	0.1217	2.2406	0.4426	0.3287	1.7884	0.3934	0.1733	2.1437	0.1475	0.4083	1.6451
2VIK	0.5820	0.0409	2.4145	0.5238	0.0690	2.4279	0.3889	0.0605	2.3855	0.8333	0.3613	1.7143
1AON	0.5333	0.0097	2.4892	0.6516	0.2539	1.9649	0.3806	0.0567	2.3742	0.5677	0.0192	2.4647
1BNI	0.4599	0.0242	2.4601	0.4722	0.0665	2.3697	0.4352	0.0383	2.4312	0.4722	0.0304	2.4478
1BRS	0.4906	0.0146	2.4764	0.5618	0.0601	2.3832	0.3371	0.0935	2.2913	0.5730	0.0165	2.4781
1BTA	0.4906	0.0146	2.4764	0.5618	0.0601	2.3832	0.3371	0.0935	3.0326	0.5730	0.0165	2.4781
1DK7	0.5274	0.0079	2.4393	0.6370	0.2384	1.4444	0.3767	0.0710	2.3424	0.5685	0.0250	2.4525

1FKB	0.5483	0.0123	2.4856	0.6168	0.0786	2.3321	0.4206	0.0772	2.4614	0.6075	0.7570	2.4235
1GXT	0.5393	0.0111	2.4847	0.6742	0.0993	2.2984	0.4045	0.0421	2.4229	0.5393	0.0353	2.4422
1HEL	0.5814	0.0354	2.4218	0.4729	0.0695	2.3508	0.5349	0.0467	2.4057	0.7364	0.2020	2.0602
1HMK	0.4242	0.0262	2.4592	0.4628	0.0428	2.4105	0.3059	0.0763	2.1671	0.5041	0.0125	2.6816
1JOO	0.3379	0.1128	2.2604	0.4830	0.1439	2.1732	0.3265	0.1475	2.1731	0.2041	0.2862	1.8529
1L63	0.3683	0.0541	2.3856	0.4877	0.0800	2.3263	0.3889	0.0396	2.4176	0.2284	0.2370	1.9633
1N88	0.6354	0.0680	2.4242	0.5938	0.1637	2.1379	0.3646	0.0772	2.3344	0.9479	0.7570	0.6615
1PGB	0.3939	0.0504	2.4240	0.4727	0.3691	2.8196	0.4000	0.1888	2.5572	0.3091	0.1862	3.0680
1PHP	0.5693	0.0210	2.4784	0.6301	0.2053	2.0571	0.3699	0.0643	2.3585	0.7078	0.1354	2.2411
1PHP-N	0.5709	0.0190	2.4868	0.6782	0.1800	2.1254	0.3736	0.0547	2.3765	0.6609	0.0904	2.3089
1QOP-1	0.5734	0.0178	2.4676	0.6455	0.0847	2.3123	0.4403	0.0506	2.3869	0.6343	0.0756	2.3339
1QOP-2	0.5819	0.0139	2.5328	0.6333	0.0969	2.2829	0.4458	0.0222	2.4507	0.6667	0.0744	2.3383
1RA9	0.5304	0.0063	2.4946	0.5912	0.0681	2.4768	0.4025	0.0291	2.4451	0.5975	0.0322	2.4435
1SCE	0.4021	0.0278	2.4484	0.6082	0.0509	2.3984	0.3505	0.0786	2.3294	0.2474	0.1842	2.1115
1UBQ	0.4211	0.0116	2.4999	0.4474	0.1114	2.2540	0.3421	0.0942	2.3613	0.4737	0.1724	1.9500
2BLM	0.4410	0.0258	2.4498	0.5615	0.1342	2.1942	0.3885	0.0618	2.3613	0.3731	0.0561	2.3737
2LZM	0.3679	0.0562	2.3812	0.4878	0.0808	2.3227	0.3841	0.0432	2.4085	0.2317	0.2330	1.9761
2RN2	0.5075	0.0108	2.4826	0.5871	0.0743	2.3356	0.4258	0.0418	2.4127	0.5097	0.0113	2.4903
2VIK"	0.5820	0.0409	2.4145	0.5238	0.0690	2.3484	0.3889	0.0605	2.3855	0.8333	0.3613	1.5831
3CHY	0.4974	0.0068	2.4968	0.5781	0.1574	2.1550	0.3359	0.0958	2.2879	0.5781	0.0708	2.3393
